# Genome-Wide Analysis Reveals Expansion and Positive Selection of Monocarboxylate Transporter Genes Linked to Enhanced Salinity and Ammonia Tolerance in *Sinonovacula constricta*

**DOI:** 10.3390/ani15060795

**Published:** 2025-03-11

**Authors:** Yiping Meng, Liyuan Lv, Hanhan Yao, Zhihua Lin, Yinghui Dong

**Affiliations:** 1 School of Marine Sciences, Ningbo University, Ningbo 315010, China; m1477684810@163.com; 2 Ninghai Institute of Mariculture Breeding and Seed Industry, Zhejiang Wanli University, Ninghai 315604, China; 3 Key Laboratory of Aquatic Germplasm Resources of Zhejiang, Zhejiang Wanli University, Ningbo 315100, China; yaohanhan1020@126.com (H.Y.); zhihua9988@126.com (Z.L.); 4 College of Advanced Agricultural Sciences, Zhejiang Wanli University, Ningbo 315101, China

**Keywords:** *Sinonovacula constricta*, monocarboxylate transporter genes, evolution, salinity challenge, ammonia nitrogen stress, gene expression

## Abstract

Our previous genome analysis of *Sinonovacula constricta* revealed an expansion of the monocarboxylate transporter gene family, which is crucial for metabolic dynamic balance and intracellular pH regulation. To further elucidate the role of these expanded *MCT* genes in response to variable environmental conditions, we conducted a comprehensive genome-wide identification, phylogenetic evolution and expression analysis. In this study, 16 sodium-coupled monocarboxylate transporter genes (designated as *ScSMCTs*) and 54 proton-coupled monocarboxylate transporter genes (designated as *ScMCTs*) were identified from the *S. constricta* genome. The results of gene number comparison indicated significant expansion of *ScSMCTs* and *ScMCTs* in mollusks compared to vertebrates, likely due to tandem repeats and dispersed duplications in *S. constricta*. The syntenic analysis demonstrated that the razor-clam *MCT* genes had the highest number of homologous gene pairs with *Meretrix meretrix*. The phylogenetic tree showed that *MCT* and *SMCT* proteins were distinctly clustered in two large branches. Moreover, positive selection analysis revealed three positive selection sites in the *MCT* amino acid sequences sites. Multi-transcriptome analyses and the temporal expression patterns displayed that *ScSMCTs* and *ScMCTs* play distinct roles in response to salinity and ammonia stressors. It is worth noting that the majority of these genes involved in abiotic stresses belong to *MCTs*. Overall, our findings revealed the important roles of *ScSMCTs* and *ScMCTs* under abiotic stress, and provided valuable information for the evolution of this family in mollusks, as well as a theoretical basis for the further study of the mechanism and function of this gene family in *S. constricta*.

## Simple Summary

Monocarboxylate transporter genes facilitate the transport of lactate, pyruvate, ketone bodies, etc., crucial for metabolic dynamic balance and intracellular pH regulation. To explore the roles of monocarboxylate transporter genes in the environmental tolerance of *Sinonovacula constricta*, a comprehensive genome-wide identification, phylogenetic evolution and expression analysis were conducted. Here, our genome-wide analysis identified 16 sodium-coupled monocarboxylate transporter genes (*ScSMCTs*) and 54 proton-coupled monocarboxylate transporter genes (*ScMCTs*) in *S. constricta*. Comparative genomic analysis revealed significant expansion of *ScSMCTs* and *ScMCTs* in mollusks compared to vertebrates, driven by tandem repeats and dispersed duplications. Transcriptome and temporal expression analyses demonstrated distinct roles of *ScSMCTs* and *ScMCTs* in response to salinity and ammonia stress, with *MCTs* playing a predominant role in abiotic stress responses. This study provides insights into the evolutionary expansion and functional divergence of monocarboxylate transporter genes in mollusks, offering a foundation for further research on their mechanisms in *S. constricta*.

## 1. Introduction

Monocarboxylates, containing 2–4 carbon atoms and a carboxyl-terminal, are important metabolites as well as energy sources for all cells in the body [1,2]. Among them, pyruvate and L-lactate are the most important, which play an important role in glucose, lipid, and amino acid metabolism [3,4]. Importantly, it has been experimentally demonstrated that the intercellular transport of monocarboxylates in their ionic form relies on monocarboxylate transporters [5,6].

So far, two types of transporters have been identified for regulating the transfer and handling of monocarboxylates: the proton-coupled monocarboxylate transporters (*MCTs*) and the sodium-coupled monocarboxylate transporters (*SMCTs*). The former belongs to the solute carrier family 16 (*SLC16*), while the latter is part of the solute carrier family 5 (*SLC5*). The primary distinction between these transporters lies in the coupling mechanisms: *MCTs* utilize the proton (H^+^) gradient to facilitate monocarboxylate transport, whereas *SMCTs* employ the sodium ion (Na^+^) gradient, thus clarifying their difference in energy requirements. The *MCT* family was first identified by Poole and Halestrap laboratories, and to date, it has included 14 members based on either sequence similarity or functional characteristics [7,8,9,10]. It is worth noting that only four members (*MCT1*, *MCT2*, *MCT3*, *MCT4*) transport monocarboxylate with a proton across the plasma membrane [10,11]. Among the other 10 members, *MCT8* has been experimentally proven to transport thyroid hormones and *MCT10* is a high-affinity aromatic amino acid transporter, with the majority of unknown substrates [12,13]. In contrast, *SMCTs* facilitate the exchange of short-chain monocarboxylate and sodium ions in a ratio of 1:2 or 1:1 [14]. This family only contains *SMCT1* (*SLC5A8*) and *SMCT2* (*SLC5A12*), with 57% identity and 73% similarity in the amino acid sequence between these two proteins [15,16,17]. Additionally, a study compared the ability of monocarboxylates to inhibit niacin uptake in the oocytes of the African clawed frog, *Xenopus laevis*, showing that *SMCT2* has a lower affinity for transport than *SMCT1* [16]. Monocarboxylate transporters have been well studied in mammals and participate in a variety of physiological processes, including promoting the absorption of nutrients, regulating cell pH, affecting metabolic homeostasis, and serving as a target for tumor therapy [2,18,19,20,21]. For example, in a study of propionate used for gluconeogenesis in the bovine liver, it was found that there are differences in *MCT1* expression levels between pre-ruminant and adult bovine livers, suggesting that *MCT1* may promote the transportation of propionate in the liver and could be regulated by development and metabolism [22,23,24]. Additionally, *MCT1* in ruminants transports SCFA into the blood by coupling with H^+^, thus reducing the risk of rumen epithelial cell acidosis and ultimately maintaining the stability of the rumen pH environment [25].

Given the significance of monocarboxylate transporters in mammalian metabolic regulation and homeostasis, studies have also been carried out in several aquatic animals [26,27,28,29,30,31,32,33,34,35,36,37,38,39,40,41]. In the largemouth bass, *Micropterus salmoides,* the expression of *MCT1* and *MCT4* was induced by hypoxic stress to cope with large amounts of lactate accumulation in tissues [31]. Similarly, a comparative analysis of the transcriptome of the hard clam, *Mercenaria mercenaria*, exposed to air for different periods revealed that about 80% of *MCT* expression levels were upregulated during air exposure, preventing lactate accumulation in cells and allowing for a high glycolysis rate and energy supply [32]. Monocarboxylate transporters have also been investigated in milkfish, *Chanos chanos,* and environmental salinities were found to impact the lactate utilization capacity under hypothermal stress [33]. Specifically, under hypothermal stress, the mRNA expressions of *MCT1* and *MCT4* were significantly upregulated in the liver and white muscle, respectively, and the abundance of *CcMCT1* and *CcMCT4* in the SW/18 °C group was higher than that in FW/18 °C group. Recently, a new *SMCT* gene (*PvSMCT1*) was identified in Pacific white shrimp, *Penaeus vannamei*, and the results uncovered that *PvSMCT1* was involved in resistance to white spot syndrome virus (WSSV) infection [34]. Furthermore, a genome-wide selective sweep analysis showed that *SMCT1* was associated with low salinity tolerance and hypotonic osmotic regulation of mefugu *Takifugu obscurus* [37]. Overall, monocarboxylate transporters profoundly affect lactate metabolism, immune regulation, and osmoregulation in aquatic organisms.

The razor clam (*Sinonovacula constricta*) is an economically important species found in intertidal zones along the coasts of southeastern China and the western Pacific Ocean. In such highly dynamic and stressful areas, osmoconforming clams can endure a wildly fluctuating temperature, pH, and salinity, as well as high ammonia nitrogen levels, and it is considered an excellent creature for exploring adaptive evolution [42]. Correspondingly, *S*. *constricta* has evolved specific adaptations to thrive in this dynamic environment [43,44,45]. One key aspect of these adaptations involves the physiological mechanisms mediated by *MCTs* and *SMCTs*. Specifically, under unsuitable salinity conditions, *MCTs* facilitate the efflux of lactate and other metabolites to prevent cellular dehydration, while *SMCTs*, driven by sodium gradients, help accumulate organic osmolytes to counteract osmotic stress; temperature variations disrupt cellular energy metabolism, leading to lactate accumulation, which *MCTs* transport out of cells to maintain the energy balance and pH stability; elevated ammonia levels interfere with the cellular pH and energy metabolism, and *MCTs*/*SMCTs* contribute to pH regulation by transporting monocarboxylates, while *SMCTs* may assist in the uptake of alternative energy substrates under ammonia stress. Importantly, a previous genome analysis of *S. consticta* uncovered the expansion of monocarboxylate transporter genes. Thus, in this study, to investigate the role of monocarboxylate transporters under environmental stresses of *S. constricta*, genome-wide identification of the *SMCT* and *MCT* gene families was performed. Subsequently, a phylogenetic tree, chromosome location, syntenic analysis, and selective pressure analysis were conducted. In addition, we probed the expression patterns of monocarboxylate transporter genes under salinity, ammonia nitrogen, and thermal stress conditions in detail. Briefly, our findings will provide a theoretical basis for the future study of the mechanism and function of the monocarboxylate transporter gene family, with significant implications for genetic improvement and enhanced environmental adaptation capacities in *S. constricta*.

## 2. Materials and Methods

### 2.1. Identification of Monocarboxylate Transporter Genes in S. constricta

The monocarboxylate transporter genes of the razor clam (designated as *ScSMCT* and *ScMCT*) were identified by searching the whole genome sequence database (PRJNA559038) using BLAST GUI Wrapper on TBtools (v1.113) with an e-value cutoff of 1 × 10^−10^ [46]. The amino acid sequences of *SMCT* and *MCT* genes in *Homo sapiens*, *Mus musculus*, *Rattus norvegicus*, *Xenopus laevis*, *Xenopus tropicalis*, and *Danio rerio* were used as a query database obtained from the NCBI (http://www.ncbi.nlm.nih.gov/, (accessed on 16 August 2024)) and ZFIN (https://zfin.org/, (accessed on 16 August 2024)). Then, the candidate genes of *ScSMCT* and *ScMCT* were verified via BLASTP against the NCBI UniProtKB/Swiss-Prot database, followed by confirming the corresponding conserved domain in the NCBI CD-search (https://www.ncbi.nlm.nih.gov/Structure/cdd/wrpsb.cgi, (accessed on 16 August 2024)) and SMART (http://smart.embl.de/smart/set_mode.cgi?NORMAL=1, (accessed on 16 August 2024)). The basic physicochemical properties of *ScSMCT* and *ScMCT*, including protein length, isoelectric point (pI), and molecular weight (MW) were determined in ExPASy (https://web.expasy.org/compute_pi/, (accessed on 17 August 2024)). Their transmembrane structures were predicted using TMHMM2.0 (https://services.healthtech.dtu.dk/services/TMHMM-2.0/, (accessed on 17 August 2024)). Furthermore, to compare the gene numbers of *SMCT* and *MCT* among species, the same identification process was applied to birds (*Gallus gallus*, *Columba livia*), amphibians (*Xenopus laevis*, *Xenopus tropicalis*), reptiles (*Trachemys scripta elegans*, *Chelonia mydas*), fish (*Ictalurus punctatus*), gastropods (*Aplysia californica*, *Lottia gigantea*), cephalopods (*Octopus sinensis*), and bivalves (*Crassostrea gigas*, *C. virginica*, *Pinctada imbricata*, *Anadara broughtonii*, *Tegillarca granosa*, *Cyclina sinensis*, *Meretrix meretrix*, *Mizuhopecten yessoensis*, *Azumapecten farreri*, *Pecten maximus*).

### 2.2. Chromosome Location and Synteny Analysis of ScSMCTs and ScMCTs

According to the Generic Feature Format (GFF) file, the distributions of *ScSMCT* and *ScMCT* genes on *S. constricta* chromosomes were exhibited by TBtools. The genes were renamed based on their placement on chromosomes from top to bottom.

After preparing the genome sequences and GFF files of *S. constricta* and the three other bivalve shellfish (*C. gigas*, *C. sinensis*, and *M. meretrix*), synteny analysis was carried out by One Step MCScanX on TBtools, and subsequently visualized by Dual Systeny plot.

### 2.3. Alignment and Phylogenetic Analysis of ScSMCTs and ScMCTs

Multiple alignments of the sequences of *SMCT* and *MCT* conserved domains from *S. constricta*, *C. gigas*, *M. yessoensis*, *T. granosa*, *C. sinensis*, *M. meretrix*, *D. rerio*, *I. punctatu.* and *H. sapiens* were performed using MAFFT with the E-INS-I algorithm. As predicted by ProtTest 3.4.2, the best-fit evolutionary model of the above aligned sequences was LG+F. The maximum likelihood (ML) phylogenetic tree was generated using IQ-Tree with 1000 bootstrap replicates. The Bayesian inference (BI) phylogenetic tree was conducted with MrBayes 3.2.6 with Markov Chain Monte Carlo (MCMC) for 10,000,000 generations. The first 25% of BI trees were discarded as burn-in, and the sampling was terminated when the convergence value was less than 0.01. The iTOL was exploited to visualize the phylogenetic tree (https://itol.embl.de/, (accessed on 25 August 2024)). The BI analyses were performed by MrBayes 3.2.6.

### 2.4. Adaptive Evolution Analysis

The site model was used to detect the positive selection of monocarboxylate transporter genes in razor clams by EasyCodeML [47]. In this model, the fitness of three pairs of nested models (M0 Vs. M3; M1a Vs. M2a; M7 Vs. M8) was compared by the likelihood ratio test (LRT). Specifically, only if the LRT passed the alternative hypothesis model (*p* < 0.05), was the gene considered to have undergone positive selection. Then, the posterior probability of the site corresponding to the ω value was calculated according to the Bayesian empirical Bayes (BEB). Positively selected sites were inferred with posterior probability of greater than 95%.

### 2.5. The Expression Profiles of ScSMCT and ScMCT Genes Based on Transcriptome Data

The expression profiles of *ScSMCT*s and *ScMCT*s were collected from the NCBI SRA database (SRR9959746–SRR9959754, SRR9943679–SRR9943690, SUB14268245), including under conditions of salinity, ammonia nitrogen, and thermal stress [48]. The expression levels of *ScMCT* and *ScSMCT* genes were evaluated in the form of TPM (transcripts per kilobase million) with the R package DESeq2 applied in R. Finally, heatmaps were generated with TBtools.

### 2.6. Stress Treatment and qRT-PCR Analysis

Adult razor clams (average shell length: 50.0 ± 2.93 mm) were collected from Ningbo Marine and Fishery Science and Technology Innovation Base in Zhejiang province, and then kept in the aerated seawater with a salinity of 25 ± 1 ppt under a temperature of 25.0 ± 1.5 °C for five days. Subsequently, three sets of experiments were performed (salinity, ammonia nitrogen, heat stress), with three biological replicates in each group. In the first set of salinity experiments, 270 clams were randomly allocated into the normal seawater group (25 ppt), low-salinity group (3 ppt), and high-salinity group (38 ppt). The water salinity of the hypotonic group and hypertonic group was gradually adjusted by 4 ppt daily until the target salinity was achieved, at which point the formal experimentation commenced. In the second set of ammonia nitrogen experiments, 120 clams were subjected to high-ammonia-nitrogen stress with a concentration of approximately 46 mg/L. Throughout the culture period, the pH was maintained at about 8 by the addition of 1.0% NaH_2_PO_4_ and 2 mg/L NaHCO_3_. For the high-temperature treatment, 240 healthy clams of uniform size were randomly transferred into the heat shock group (32 °C) and the control group (25 °C). The temperature of the treatment group was gradually elevated at a rate of 4 °C per hour until the target temperature was reached. At various time points post-challenge (0 h, 3 h, 6 h, 12 h, 24 h, 48 h, and 72 h), the gills were isolated from six randomly sampled clams. All samples were promptly snap-frozen in liquid nitrogen and stored at −80 °C after collection.

Total RNA was extracted from the gills by using Trizol (Sangon, Shanghai, China) and then cDNA was synthesized by 1.5 μg RNA with the Prime-Script™ RT reagent kit (TaKaRa, Kusatsu, Japan) in accordance with the manufacturer’s instructions. The real-time PCR primers for monocarboxylate transporter genes were designed by Primer 5 software (Table 1). The *RS9* gene served as an internal reference. The iTaq^™^ universal SYBR^®^ Green Supermix (Bio-Rad, Hercules, CA, USA) was employed in the LightCycler^®^ 480II (Roche, Pleasanton, CA, USA). All samples were performed with six biological parallels and three technical replicates. The expression levels of monocarboxylate transporter genes were quantified using the method of 2^−△△CT^, and then processed in GraphPad Prism 8.0. Statistical analysis of the differences between groups was conducted using a one-way analysis of variance (one-way ANOVA) with SPSS 21.0 software.

## 3. Results

### 3.1. Expansion Analysis of ScMCT and ScSMCT Genes from Representative Vertebrates and Invertebrates

Sixteen *ScSMCT* and fifty-four *ScMCT* genes were identified from the genome of *S. constricta*. The basic physicochemical properties of *ScSMCT* and *ScMCT* genes are shown in Table S1. Briefly, the amino acids residues of *ScSMCT* and *ScMCT* proteins are from 371 to 788, with the putative molecular weights from 18.280 kDa to 85.476 kDa, and isoelectric points (pIs) from 5.45 to 9.62. The result of subcellular localization revealed that almost all proteins were localized to the cell membrane, except for *ScSMCT2_7*, which was found in the nucleus.

**Figure 1:**
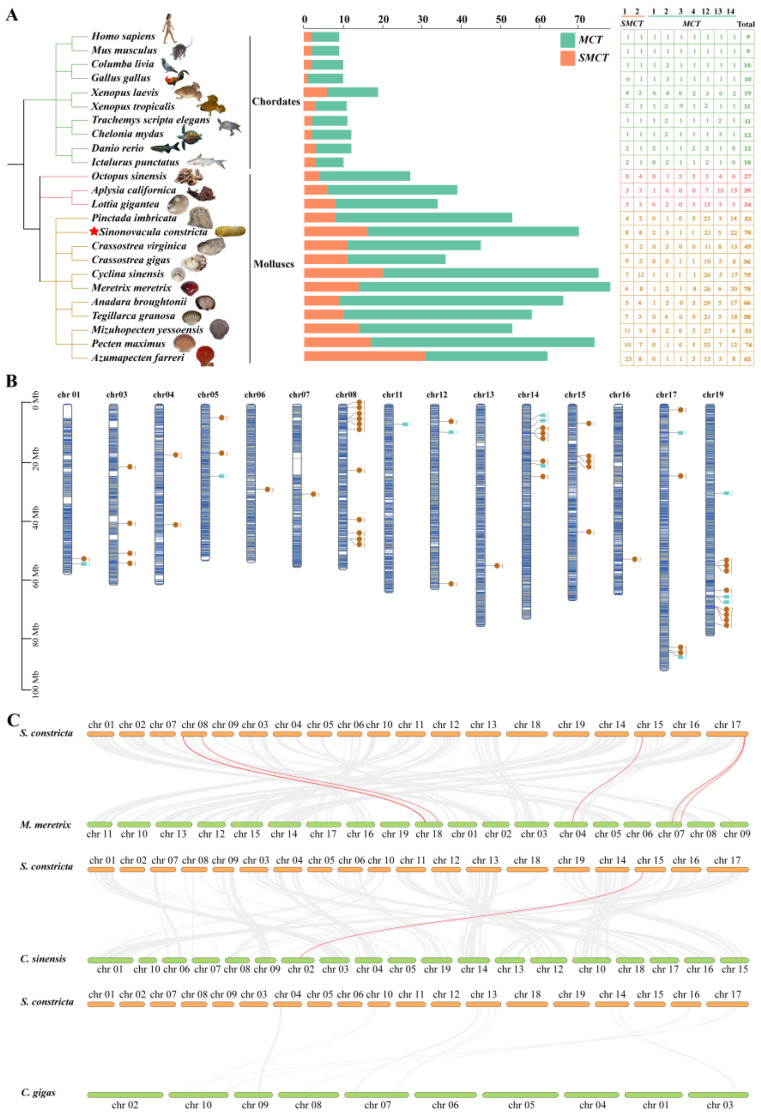
Expansion and comparative analysis of *MCTs* and *SMCTs* in *S. constricta* and other species. (**A**): the numbers of *SMCT* and *MCT* genes in *S. constricta* and other representative species. Except for *H. sapiens* (https://www.britannica.com, (accessed on 19 January 2025)), *O. sinensis*, and *C. virginica* (https://www.ncbi.nlm.nih.gov/, (accessed on 19 January 2025)), the images of the remaining species are sourced from https://www.inaturalist.org (accessed on 19 January 2025). The red star represented the target species in this study. (**B**): The distribution of 70 *MCTs* and *SMCTs* on the razor-clam chromosomes. The blue squares and brown circles represented the *ScSMCTs* and *ScMCTs*, respectively. (**C**): Synteny analysis of monocarboxylate transporter genes between *S. constricta* and other bivalves (*C. gigas*, *C. sinensis*, and *M. meretrix*). Gray lines denote the collinear blocks, while the collinear gene pairs are highlighted by the red lines.

The numbers of monocarboxylate transporter genes in razor clams and selected species (including mollusks, fish, amphibians, reptiles, birds, and human) were determined and are exhibited in Figure 1A. In general, except for *X. laevis* (up to 6siz), *G. gallus* (up to three) and *C. mydas* (up to three), the *SMCT* and *MCT* genes exist as single copies in mammalian genomes and typically no more than two copies in fish. However, the significantly increased copy numbers of monocarboxylate transporter genes were found in mollusks, especially bivalves, ranging from 36 to 78.

### 3.2. Chromosomal Location, Synteny, and Phylogenetic Evolution of ScSMCT and ScMCT Gene Families

The monocarboxylate transporter genes were unevenly mapped on 15 chromosomes through the available genomic data and annotation information of *S. constricta* (Figure 1B). The results reflected that chromosome 19 contained the most genes (15), followed by chromosome 14 (12), chromosome 8 (11), chromosome 17 (6), chromosome 15 (5), and chromosome 3 (4), whereas chromosomes 4/5/12 had three genes, and only one monocarboxylate transporter gene and two genes were distributed on chromosomes 6/7/11/13 and chromosome 1/16, respectively. In addition, about 35.7% of these genes may have been involved in tandem duplication.

To investigate the phylogenetic mechanism of the monocarboxylate transporter genes, the syntenic analysis was performed in four bivalve species (*S. constricta*, *C. gigas*, *C. sinensis*, and *M. meretrix*). The results showed that six collinear gene pairs between *S. constricta* and *M. meretrix* were identified, followed by *S. constricta and C. sinensis* (one orthologous gene pair on Chr15) (Figure 1C), and these orthologous genes all belong to *MCT* gene family. Moreover, one *ScMCT* gene was associated with only one syntenic gene pair, indicating that *MCT* genes in *S. constricta*, *C. sinensis*, and *M. meretrix* evolved from the same ancient *MCT* genes. These results suggested that *MCT* genes might play an important role in the monocarboxylate transporter family evolution, and the razor-clam *MCT* genes were closer to the *M. meretrix* genes than to *C. sinensis* and *C. gigas* genes.

**Figure 2:**
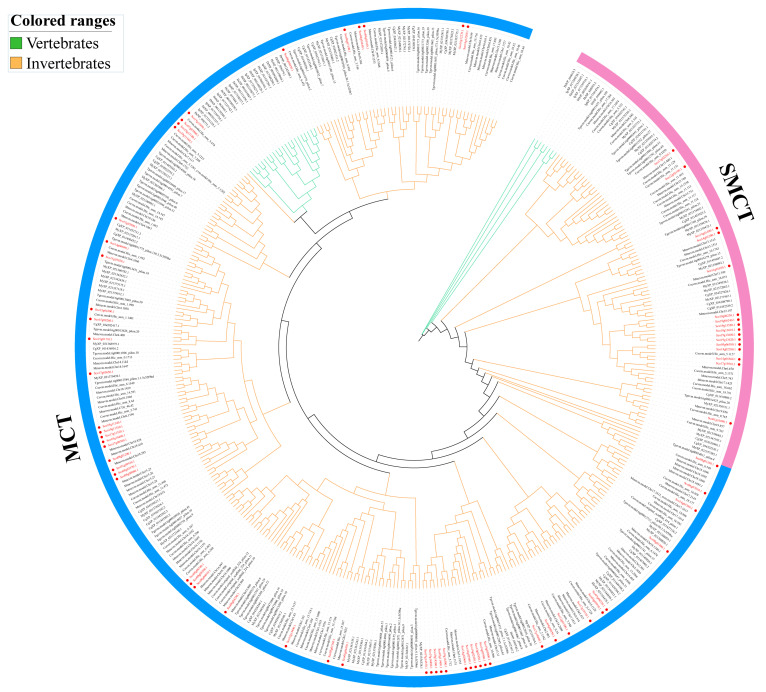
Phylogenetic tree of the amino acid sequences of monocarboxylate transporter proteins. The tree is constructed by the maximum likelihood algorithm using the IQ-Tree software based on the multiple sequence alignment by MAFFT. The reliability of the branching was tested by bootstrap re-sampling (1000 pseudo-replicates). The monocarboxylate transporter proteins of *S. constricta* are represented by a red circular symbol.

To explore the phylogenetic relationship among the monocarboxylate transporter genes in *S. constricta*, a phylogenetic tree was constructed by IQ-Tree with 1000 bootstraps. In addition, the topological structure of the phylogenetic tree established by Bayesian inference is basically identical to that of the former, indicating that the phylogenetic tree results are credible (Figure S1). As is shown in Figure 2, the gene family was clearly clustered, that is, *MCT* and *SMCT* proteins were clustered in two large branches. This reveals that although the proton-coupled monocarboxylate transporter family and the sodium-coupled monocarboxylate transporter family are functionally similar, they have also undergone significant differentiation during evolution. In addition, individual members of *S. constricta* were clustered with vertebrates, such as *ScMCT12_21* (Sco19g15890.1), which was found to be in the same clade with vertebrate *MCTs*, while most of them were clustered in the mollusk group, indicating that the *MCT* and *SMCT* gene families may have lineage-specific expansion.

By codon-based site tests in EasyCodeML, the amino acid sites under positive selection were further identified using the BEB approach. As is illustrated in Table 2, comparisons of M0 (one-ratio) vs. M3 (discrete) revealed statistical significance, indicating variable selection pressure among codons across the *SMCT* and *MCT* subfamilies. Obviously, only M7 (beta)/M8 (beta and ω) comparison pairs detected the significant results (*p* < 0.05), which provided the evidence of positive selection for *SMCT* and *MCT* subfamilies. Furthermore, there are three positive selection sites (2 V 0.998, 13 I 0.997, 14 A 1.000) in the MCT amino acid sequences, and two of them (13 I 0.997, 14 A 1.000) were located in the protein functional domain, indicating that positive selection may play an important role in driving the *MCT* protein evolution in *S. constricta* (Figure S2). Although some sites on the *SMCT* genes are subject to positive selection, the information or evidence for each individual site is too weak (439 G 0.514, 475–0.749). These results suggest that the *MCT* and *SMCT* subfamilies have different molecular evolutionary patterns in the face of dynamic environmental changes.

### 3.3. Transcriptome Analysis Links Multiple ScSMCTs and ScMCTs to Salinity and Ammonia Nitrogen Stress in the Gill Tissue

Based on the available transcriptome data, the expression patterns of all 70 monocarboxylate transporter genes were analyzed under conditions of salinity, ammonia nitrogen and heat stress to confirm whether *ScSMCTs* and *ScMCTs* are involved in the response to various stresses. We observed distinct gene expression profiles following various stimuli and differential gene responses to various stresses.

**Figure 3:**
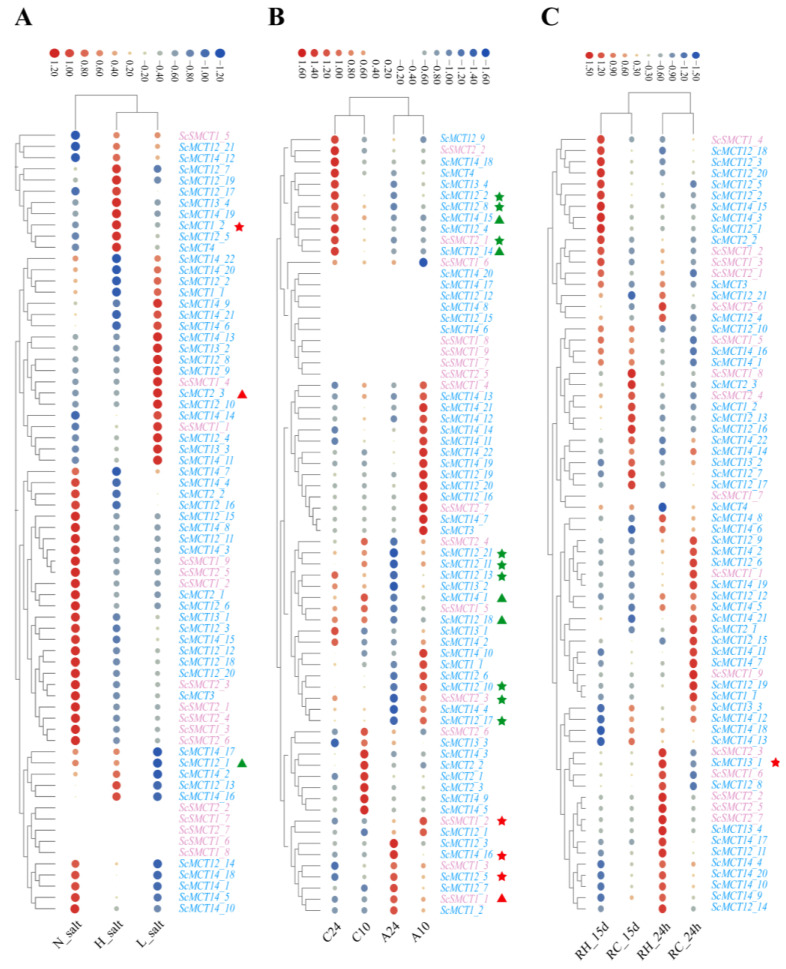
Expression patterns of *ScMCT* and *ScSMCT* in response to salinity (**A**), ammonia nitrogen (**B**), and heat stress (**C**). The *MCT* genes were represented by blue, and the *SMCT* genes were represented by pink. N_salt: normal salinity (25 psu), H_salt: high salinity (38 psu), L_salt: low salinity (3 psu). C24 and A24: stress at 0.31 mg/L and 180 mg/L ammonia nitrogen concentration for 24 h, respectively; C10 and A10: stress at 0.31 mg/L and 180 mg/L ammonia nitrogen concentration for 10 d, respectively. RC and RH represented the control and thermal-stress groups, respectively. The symbols adjacent to the gene name represent significantly differentially expressed genes, and upregulated and downregulated genes are illustrated by red and green colors. The stars (★) and triangles (▲) in the A plot represent significant differential genes under low-salt and high-salt conditions, respectively. In the B and C plots, these symbols correspond to differentially expressed genes under short-term and long-term stress, respectively.

As is illustrated in Figure 3A, compared with the control group, only one and two differentially expressed *ScSMCT* and *ScMCT* transcripts were detected under high- and low-salinity stress, respectively. Specifically, the expression level of *ScMCT1_2* was considerably upregulated under hyperosmotic stress (*p* < 0.05). Additionally, in response to a hypotonic environment, another *MCT* gene (*ScMCT2_3*) was significantly upregulated, while *ScMCT12_1* exhibited the opposite expression trend (*p* < 0.05) (Figure 3A).

In contrast to the limited number of genes responding to salinity stress, more genes (17 in total) demonstrated altered expression levels following exposure to ammonia nitrogen stress (Figure 3B). However, downregulated genes made up a significant portion of these 17 genes, indicating that they were not closely associated with the transport of metabolites such as pyruvate and lactate. Only *ScSMCT1_1*, *ScSMCT1_2*, *ScMCT12_5* and *ScMCT14_16* were significantly upregulated (*p* < 0.05). Moreover, *ScSMCT1_1* was notably influenced by both acute and chronic ammonia nitrogen stress among the genes that were upregulated.

The transcriptomic data under high temperature displayed that the expression levels of *ScSMCT* and *ScMCT* transcripts in the gill remained stable and almost unaffected by an elevated temperature (Figure 3C). Only *ScMCT13_1* gene were significantly upregulated, which was confined to the acute phase.

### 3.4. Expression Patterns of ScMCT and ScSMCT Genes in Response to Abnormal Salinity, High Ammonia Nitrogen, and Thermal Stimulation in the Gill Tissue

To investigate the expression patterns of monocarboxylate transporter genes of *S. constricta* under salinity acclimation, high-ammonia-nitrogen stress, and thermal stimulation, seven upregulated *ScMCTs* and *ScSMCTs* were evaluated using qRT-PCR.

**Figure 4:**
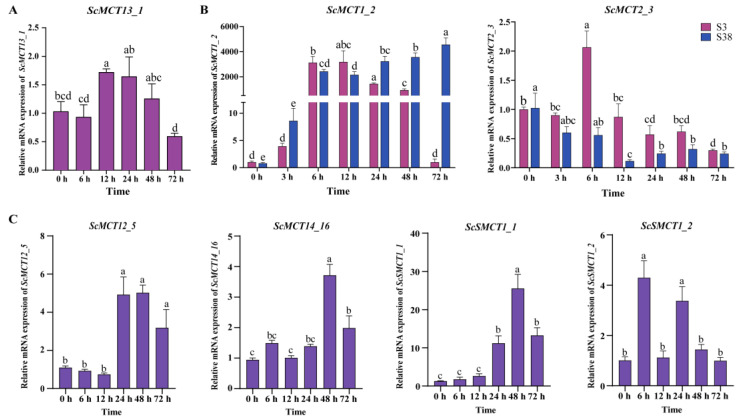
Time-dependent expression patterns of seven monocarboxylate transporter genes post-thermal-stress (**A**), salinity stimulation (**B**), and ammonia nitrogen challenge (**C**) in gills. The magenta and blue bars represent low-salinity (S3) and high-salinity (S38) stimulation, respectively. Groups of different significance levels were indicated by different letters (*p* < 0.05). Vertical bar represents mean ± SD (*n* = 3).

Examining the expression profiles of the *ScMCT13_1* gene post-thermal-stress at multiple time points, the expression levels exhibited a mild upward trend at 12 h (Figure 4A). For salinity stress, the expression levels of *ScMCT1_2* and *ScMCT2_3* demonstrated an initial upregulation followed by a downregulation in response to hypotonic stress, while exhibiting an opposite pattern under hypertonic stress (Figure 4B). Specifically, during hypotonic stress, *ScMCT1_2* reached its maximum expression at 12 h, while *ScMCT2_3* peaked at 6 h. Conversely, in the hypertonic-stress group, the transcripts of *ScMCT1_2* progressively increased, attaining the highest level at 72 h, whereas the expression levels of *ScMCT2_3* remained below the baseline at 0 h and reached a nadir at 12 h. It is noteworthy that salinity stimulation more prominently induced the upregulation of mRNA expression for *ScMCT1_2* compared to *ScMCT2_3*. During an acute ammonia nitrogen challenge, the expression of *ScMCT12_5*, *ScMCT14_16,* and *ScSMCT1_1* was significantly upregulated in the relatively late stage, while *ScSMCT1_2* began to respond in a relatively early stage (Figure 4C). Specifically, compared with 0 h, the expression levels of *ScMCT12_5* and *ScSMCT1_1* were significantly elevated at 24 h, 48 h, and 72 h, with the peak expression occurring at 48 h. Additionally, compared to 0 h, *ScMCT14_16* exhibited a significant rise at 48 h and 72 h, and showed the highest expression at 48 h. In comparison, the relative abundance of *ScSMCT1_2* was significantly upregulated at 6 h, followed by another increase at 24 h after a notable decline at 12 h. Overall, compared with temperature stress, the expression level of *ScMCTs* and *ScSMCTs* changed more dramatically under salinity and ammonia nitrogen challenges.

## 4. Discussion

The importance of monocarboxylate transport across the plasma membrane by *MCTs* and *SMCTs* in mammalian metabolic regulation and homeostasis is well established [4,20,21,49]. Recently, in aquatic animals, studies have shown that monocarboxylate transporter genes could respond to various abiotic and biotic stresses, including hypoxic, hypothermal, and hypotonic stresses, and viral infections [31,32,33,34,37]. However, the evolution of the monocarboxylate transport gene family at the genomic level has not been well elucidated in marine mollusks. In this study, a comprehensive analysis of the *SMCT* and *MCT* gene families was performed in the razor clam.

A total of 16 *SMCT* genes and 54 *MCT* genes were identified from the genome data of *S. constricta*. Collectively, these 70 monocarboxylate transporters are basically located on the plasma membrane, except for one *SMCT* gene (*ScSMCT2_7*) located on the nuclear membrane, indicating potential functional disparities. This phenomenon has been reported across multiple gene families [50,51]. For instance, members of the ATG8 gene family localized in the cytoplasm facilitate autophagosome formation, while certain nuclear ATG8 homologs (RB1CC1) are involved in distinct biological processes, such as transcriptional regulation [50]. Additionally, a gene family that consists of two or more copies from gene duplication or doubling exhibits analogous structures and functions [52,53]. Consistently, the phylogenetic tree was predominantly divided into two major clades that correspond with the functional classifications of monocarboxylate transporter genes. Among each *SLC* subfamily, several *ScSLCs* were found to cluster alongside *SLCs* from both human and fish species, while others formed a distinct group specific to bivalves, indicating that *MCTs* and *SMCTs* were highly conserved in bivalves and these genes were derived from lineage-specific events. Notably, the lineage-specific expansion of paralogous gene families, which in certain instances constitutes a substantial portion of the genome, is regarded as one of the primary mechanisms of adaptation [54,55].

The number of monocarboxylate transporter genes varies between selected vertebrates and mollusks. Unlike only a single copy of mammalian *MCT* and *SMCT* subfamily members, almost all members are multiple copies in mollusks, although the loss of individual family members often occurs in mollusks. This similar phenomenon was also found in scallop SLC family member identification and oyster TRP family member analysis [40,56]. It is worth noting that *MCT14* had 22 copies in *S. constricta*, which is the largest number of species selected. Multiple copies of genes in oysters may be related to repeated events during evolution, and duplicate genes in bony fish may be derived from WGD events [57,58,59]. Then, from the perspective of razor-clam chromosome localization, the *ScSMCTs* and *ScMCTs* are distributed on different chromosomes, and some are in a tandem array on chromosomes. These different modes of duplication contributed to this gene family expansion in razor clams. In addition, previous studies have shown that positive selection is considered to be a marker of evolutionary change and molecular adaptation [40,60]. In order to understand the evolutionary adaptation of *ScSMCTs* and *ScMCTs* in the dynamic aquatic environment, the site model was used for positive selection analysis. The results showed that three sites of *ScMCTs* were under purification selection, and two of them were located in the protein functional domain, indicating that positive selection may play an important role in driving the *MCT* protein evolution in *S. constricta.*

The gill, an important respiratory and excretory organ of bivalves, responds to various stimulations and facilitates osmotic regulation and ion regulation [61,62,63]. Additionally, respiratory metabolism, an important bioenergy process of the body, is often disturbed by environmental stresses, making the transition from aerobic respiration to anaerobic respiration [64,65,66,67,68,69,70]. Consequently, as the end product of glycolysis under hypoxic metabolism, lactate must be carried out of the cell to avoid accumulation, resulting in a reduction in the cytosol pH. In this process, *MCTs* and *SMCTs* are instrumental in the transportation of lactate [1,14]. And, some studies have reported that monocarboxylate transporter genes participated in the stress response of aquatic organisms [29,31,32]. When facing hypoxia stress, about 80% of *MCTs* in hard clams were upregulated and the expression profiles of *MCT1* and *MCT4* were induced in *M. salmoides* [31,32]. Three members of the *SLC16* family genes (*SLC16A4*, *SLC16A9A* and *SLC16A7*) in *Tenualosa ilisha* were found to be differentially expressed under diverse salinity conditions [39]. The mRNA expression level of *MCT12* (*MCT12_1*, *MCT12_2*, and *MCT12_3*) in *C. ariakensis* significantly changed post-high-salinity-challenge [71]. Similarly, in this study, based on salinity, ammonia-nitrogen, and heat-stress RNA-Seq data of the gill tissue, two, four, and one monocarboxylate transporter genes were found to be considerably upregulated, respectively, indicating their potential role in adapting to different salinity, high-ammonia-nitrogen, and high-temperature environments. Further, seven *ScMCT* and *ScSMCT* genes exhibited different expression patterns for 72 h. In the salinity treatment group, unlike *ScMCT2_3*, which was only upregulated in hypotonic stress, the expression of *ScMCT1_2* was significantly upregulated under both hypotonic and hypertonic stress, suggesting the importance of *ScMCT1_2* in euryhaline adaptation. Notably, after exposure to high concentrations of ammonia nitrogen, aquatic animals are forced to adjust their metabolic process and are faced with ammonia nitrogen accumulation [65,72]. In the ammonia treatment group, the relative abundance of *ScSMCT1_2* began to respond in the early stage and was upregulated twice during the ammonia nitrogen treatment, revealing that *ScSMCT1_2* may participate in the transport of lactate and NH_4_^+^ after the acute response phase. Obviously, after the stresses of abnormal salinity and ammonia nitrogen, the induced expression of monocarboxylate transporter genes in *S. constricta* indicated the existence of anaerobic respiration. The transport of lactate is not only conducive to the dynamic balance of metabolism, but also maintains a high glycolysis rate, ensuring a continuous energy supply. Nevertheless, the expression levels of the *MCT* gene remained relatively stable over time following exposure to high-temperature stress, implying that monocarboxylate transporter genes did not have predominant roles in response to such a stress condition. There was no overlap in the differentially expressed transcripts among the aforementioned challenge groups, suggesting that the monocarboxylate transporter genes of *S. constricta* serve distinct functions in response to diverse abiotic stressors. Furthermore, most of the monocarboxylate transporter genes with altered expression belong to *MCTs*, indicating that they are more important than *SMCTs* in stress response. Overall, our results have provided evidence that monocarboxylate transporter genes may be involved in lactate transport in the gills of the razor clam after exposure to stresses.

## 5. Conclusions

In summary, a total of 70 monocarboxylate transporter genes were identified from the genome of *S. constricta*, which were divided into two categories, namely *ScSMCTs* and *ScMCTs*. The phylogenetic tree analysis showed that monocarboxylate transporter genes were highly conserved in bivalves. In addition, monocarboxylate transporter genes were significantly expanded in mollusks. The expansion of these genes in the razor clam may be related to tandem repeats and dispersed duplications. In particular, the expression changes of monocarboxylate transporter genes in the gills of *S. constricta* under salinity and ammonia nitrogen stresses indicated that they played an important role in maintaining metabolic balance.

## Figures and Tables

**Table 1:** Sequences of the primers used in the study.

Primer Name	Sequence (5′-3′)
ScMCT1_2F	GCTCACATCACCGAAGGTCAAAT
ScMCT_2R	TCGTCACATCTTGATCCTGCT
ScMCT2_3F	AGGAGAGCGATGTTTACTGGTG
ScMCT2_3R	GCTGAATGGAACCTGGGAGT
ScMCT12_1F	TTTGCTCCTATTATGACGACT
ScMCT12_1R	TTGGCCTCATTAAAGATCCAC
ScMCT12_5F	AGGAAGGAGCCTCACCTACAC
ScMCT12_5R	GTTGCTATGCCAACAGCCA
ScMCT13_1F	ACATTCTCAGTGTCTTCGCAAC
ScMCT13_1R	GACGGTAGAAACACCATCCCAA
ScMCT14_16F	ACATCACCTAAAGGCAGCTGT
ScMCT14_16R	AGGTACTCATCTGCACACGTT
ScSMCT1_1F	CGGATAATCGTGCCCCTCA
ScSMCT1_1R	GGTCCAAATAGTACAACACCCAT
ScSMCT1_2F	GCCATTACCAACAACCGGAT
ScSMCT1_2R	CAGTCGACCCAGATAAATAAGAGC
RS9F	TGAAGTCTGGCGTGTCAAGT
RS9R	CGTCTCAAAAGGGCATTACC

**Table 2:** Site model analysis for the monocarboxylate transporter genes. LRT is likelihood ratio test statistic for M0 vs. M3; M1a vs. M2a; M7 vs. M8 (*p* < 0.05 means accepting the alternative models). Posterior probability (Pr) of greater than 95% was inferred as positive selection site.

Name of Gene Family	Number of Copies	LRT *p*-Value (Number of Positive Sites Pr > 95%)		
		M0 vs. M3	M1a vs. M2a	M7 vs. M8
*SMCT*	16	0.0000	1.0000 (0)	0.0014 (0)
*MCT*	54	0.0000	1.0000 (0)	0.0000 (3)

## Data Availability

All of the data generated or analyzed in this study are included in this paper.

## Supplementary Materials

The following supporting information can be downloaded at: https://www.mdpi.com/article/10.3390/ani15060795/s1, Table S1: Predicted physicochemical properties of 70 monocarboxylate transporter proteins in *S. constricta*; Figure S1: Phylogenetic tree of the amino acid sequences of monocarboxylate transporter proteins using BI analysis. The monocarboxylate transporter proteins of *S. constricta* are represented by a red asterisk symbol; Figure S2: The three positive selected amino acid sites in Sc*MCTs* using site models, indicated by red triangles.
