# Supplementary material for: Genome-Wide Analysis Reveals Expansion and Positive Selection of Monocarboxylate Transporter Genes Linked to Enhanced Salinity and Ammonia Tolerance in Sinonovacula constricta
Source: Animals (Basel). 2025 Mar 11;15(6):795. doi: 10.3390/ani15060795 (PMC11939762; doi:10.3390/ani15060795)
Supplement: Supplementary file 1 [file animals-15-00795-s001.zip › animals-3516481-supplementary.pdf]

animals

MDPI

Table S1. Predicted physicochemical properties of 70 monocarboxylate transporter proteins in *S. constricta.*

Gene ID	Gene Rename	Amino Acids	MW (kDa)	pI	GRAVY	Number of TMDs
Sco11g01650.1	ScSMCT1_1	638	65.65	9.07	0.48	13
Sco14g01490.1	ScSMCT1_2	618	53.28	8.76	0.55	12
Sco14g01500.1	ScSMCT1_3	628	69.67	6.83	0.50	13
Sco14g02280.1	ScSMCT1_4	596	69.38	9.08	0.60	13
Sco14g05560.1	ScSMCT1_5	627	63.06	8.87	0.57	13
Sco17g19510.1	ScSMCT1_6	623	67.46	8.75	0.66	13
Sco19g13600.1	ScSMCT1_7	495	71.23	9.13	0.58	9
Sco19g13610.1	ScSMCT1_8	604	65.21	9.02	0.59	13
Sco19g13620.1	ScSMCT1_9	604	65.24	8.90	0.61	13
Sco01g10490.1	ScSMCT2_1	655	18.28	6.26	0.51	13
Sco05g06380.1	ScSMCT2_2	599	65.71	8.00	0.55	13
Sco12g02190.1	ScSMCT2_3	631	68.56	8.52	0.59	13
Sco17g02250.1	SCSMCT2_4	499	67.96	8.46	0.28	8
Sco19g06230.1	ScSMCT2_5	584	54.97	9.24	0.61	12
Sco19g06240.1	ScSMCT2_6	601	68.85	8.88	0.64	13
Sco19g13580.1	ScSMCT2_7	597	64.90	9.23	0.62	13
Sco08g08060.1	ScMCT1_1	534	58.02	6.33	0.32	12
Sco13g11520.1	ScMCT1_2	615	65.88	5.52	0.33	12
Sco03g03910.1	ScMCT2_1	479	52.59	6.67	0.45	12
Sco03g08230.1	ScMCT2_2	451	49.43	6.65	0.52	10
Sco08g00010.1	ScMCT2_3	463	50.22	8.55	0.61	12
Sco17g19310.1	ScMCT3	440	48.82	8.46	0.52	12
Sco05g04430.1	ScMCT4	550	59.60	7.87	0.38	12
Sco04g03430.1	ScMCT12_1	628	68.69	9.16	0.28	11
Sco04g03440.1	ScMCT12_2	639	69.87	8.82	0.27	11
Sco04g08050.1	ScMCT12_3	471	50.57	8.69	0.63	11
Sco05g01160.1	ScMCT12_4	371	39.24	8.30	0.94	11
Sco07g05800.1	ScMCT12_5	658	72.65	7.59	0.04	10
Sco08g00430.1	ScMCT12_6	517	56.06	7.48	0.62	12
Sco08g01400.1	ScMCT12_7	457	50.62	7.46	0.65	12
Sco08g04230.1	ScMCT12_8	490	52.93	8.20	0.66	10
Sco08g07190.1	ScMCT12_9	624	68.40	8.78	0.17	10
Sco08g08500.1	ScMCT12_10	615	68.20	8.69	0.18	10
Sco08g08540.1	ScMCT12_11	449	49.70	7.06	0.73	11
Sco12g01390.1	ScMCT12_12	464	51.45	9.62	0.19	8
Sco12g15490.1	ScMCT12_13	472	50.50	5.96	0.75	12
Sco15g08260.1	ScMCT12_14	547	58.71	5.45	0.29	10
Sco16g12260.1	ScMCT12_15	440	48.11	8.42	0.74	12
Sco16g12270.1	ScMCT12_16	440	48.43	8.77	0.65	12
Sco17g00300.1	ScMCT12_17	622	68.67	8.70	0.32	12
Sco17g18860.1	ScMCT12_18	461	49.64	7.55	0.63	12
Sco19g11160.1	ScMCT12_19	600	65.12	8.68	0.32	12
Sco19g13180.1	ScMCT12_20	570	62.19	8.18	0.24	12
Sco19g15890.1	ScMCT12_21	647	71.94	8.85	-0.01	12
Sco01g09930.1	ScMCT13_1	544	58.39	6.05	0.48	12

Sco06g07010.1	ScMCT13_2	417	45.20	8.75	0.86	12
Sco08g01160.1	ScMCT13_3	490	53.44	8.52	0.59	12
Sco14g05380.1	ScMCT13_4	444	48.24	6.98	0.30	9
Sco03g10480.1	ScMCT14_1	481	52.28	6.59	0.65	12
Sco03g11400.1	ScMCT14_2	430	46.93	8.77	0.70	10
Sco08g01290.1	ScMCT14_3	569	61.97	5.90	0.26	11
Sco08g01970.1	ScMCT14_4	455	49.43	8.80	0.60	12
Sco14g02930.1	ScMCT14_5	494	53.66	7.97	0.52	9
Sco14g02940.1	ScMCT14_6	510	54.68	8.44	0.58	11
Sco14g02950.1	ScMCT14_7	541	58.21	7.53	0.54	11
Sco14g02960.1	ScMCT14_8	541	58.12	7.53	0.54	11
Sco14g02980.1	ScMCT14_9	456	49.46	8.28	0.50	10
Sco14g03000.1	ScMCT14_10	503	54.26	6.15	0.60	11
Sco14g06690.1	ScMCT14_11	596	64.82	7.91	0.34	11
Sco15g01710.1	ScMCT14_12	567	62.08	7.51	0.37	12
Sco15g03890.1	ScMCT14_13	651	70.24	6.14	0.10	8
Sco15g03920.1	ScMCT14_14	721	79.41	8.88	0.17	12
Sco15g04300.1	ScMCT14_15	581	63.83	8.86	0.31	12
Sco17g05650.1	ScMCT14_16	733	80.84	6.96	0.02	12
Sco19g11340.1	ScMCT14_17	417	44.85	8.23	0.66	11
Sco19g11350.1	ScMCT14_18	417	44.96	8.36	0.83	11
Sco19g14480.1	ScMCT14_19	449	48.74	8.20	0.81	12
Sco19g14500.1	ScMCT14_20	590	63.66	8.38	0.74	16
Sco19g14550.1	ScMCT14_21	788	85.48	8.63	0.76	20
Sco19g14560.1	ScMCT14_22	699	75.73	8.67	0.75	17

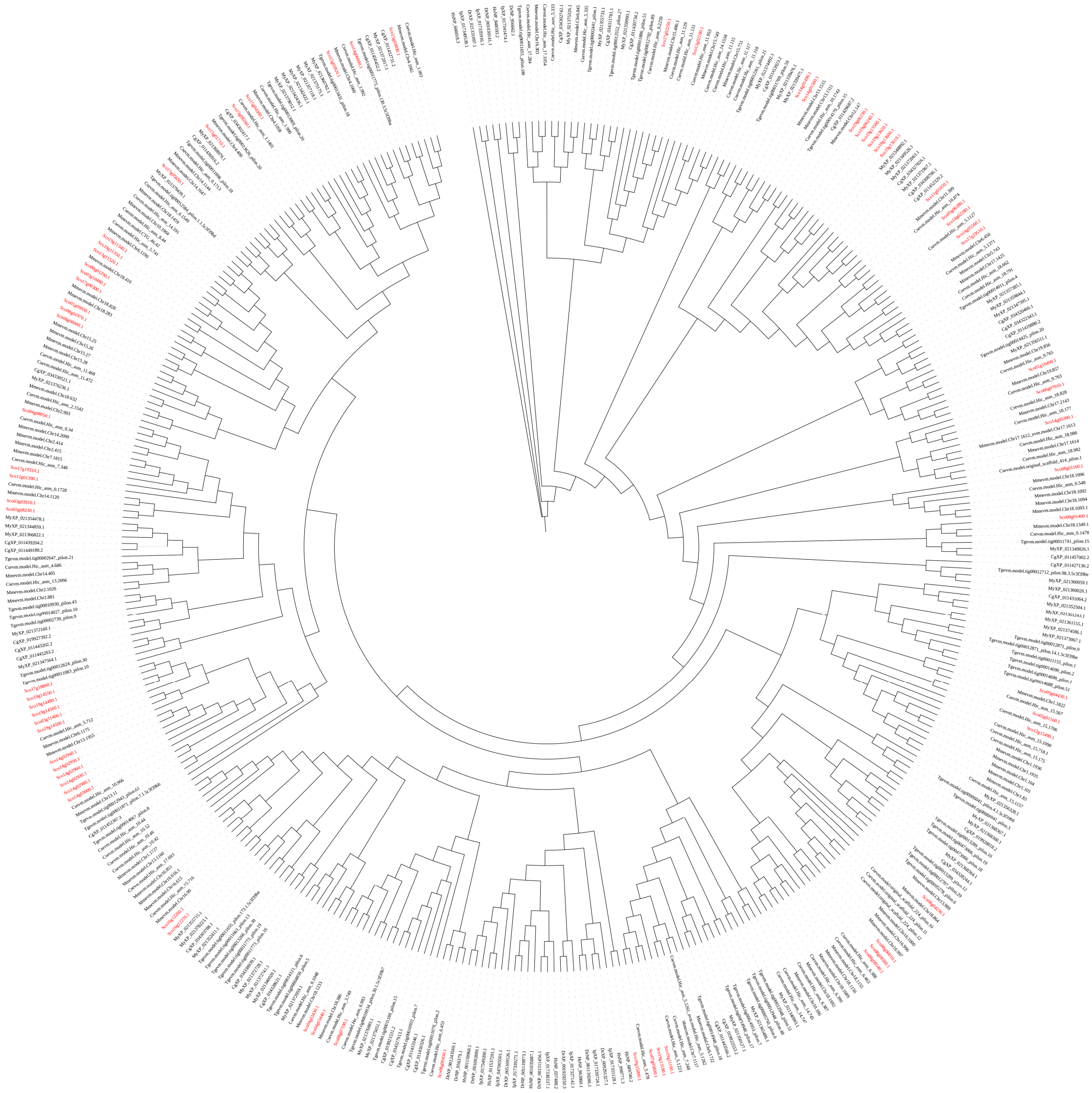

Figure S1. Phylogenetic tree of the amino acid sequences of monocarboxylate transporter proteins using BI analysis. The monocarboxylate transporter proteins of *S. constricta* are represented by a red asterisk symbol.

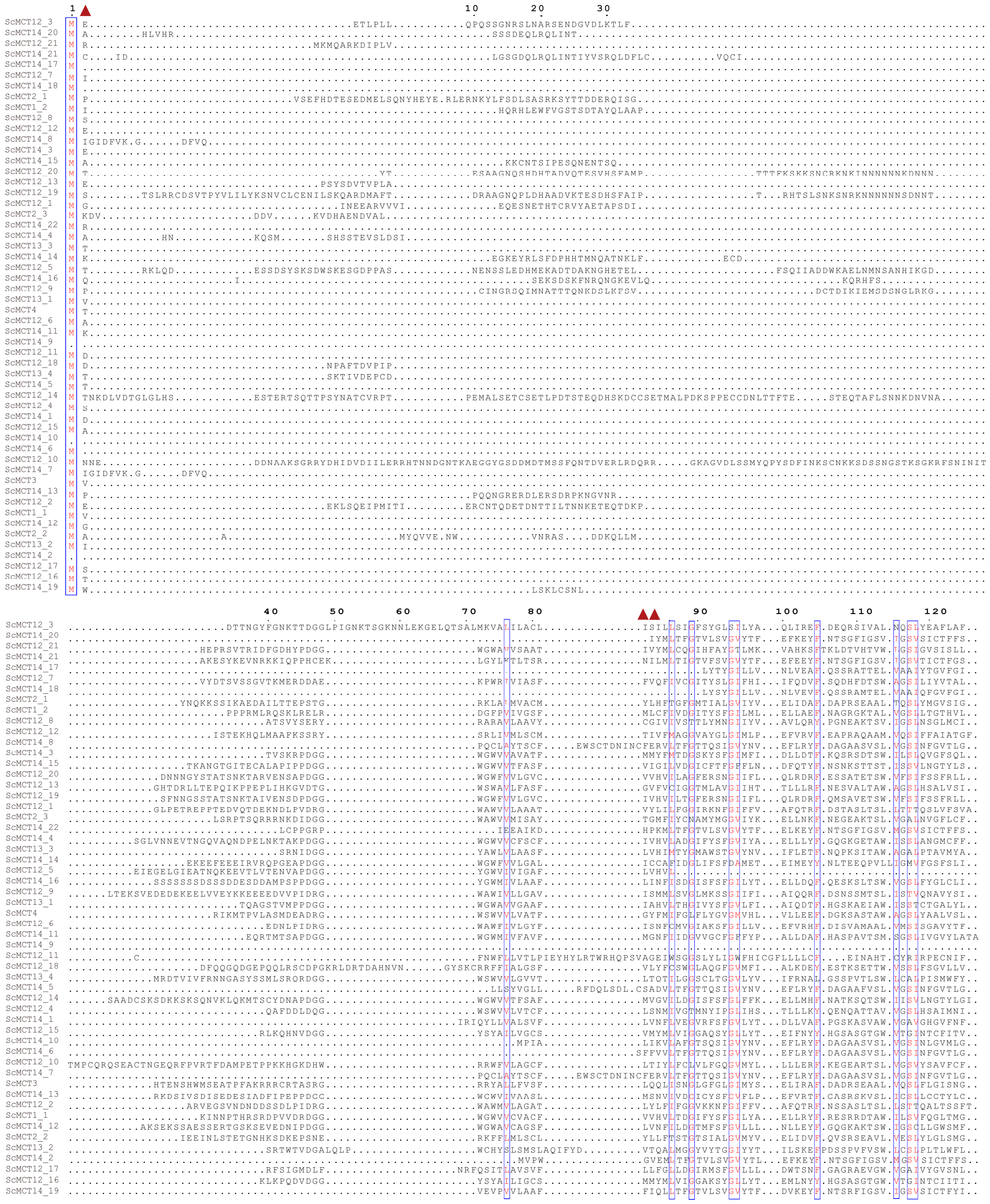

Figure S2. The three positive selected amino acid sites in Sc*MCTs* using site models, indicated by red triangles.
